# Prevalence of Bacterial Meningitis Among Febrile Infants Aged 29-60 Days With Positive Urinalysis Results: A Systematic Review and Meta-analysis

**DOI:** 10.1001/jamanetworkopen.2021.4544

**Published:** 2021-05-12

**Authors:** Brett Burstein, Vikram Sabhaney, Jeffrey N. Bone, Quynh Doan, Fahad F. Mansouri, Garth D. Meckler

**Affiliations:** 1 Division of Pediatric Emergency Medicine, Department of Pediatrics, Montreal Children's Hospital, McGill University Health Centre, Montreal, Quebec, Canada; 2 Department of Epidemiology, Biostatistics, and Occupational Health, McGill University, Montreal, Quebec, Canada; 3 Division of Pediatric Emergency Medicine, Department of Pediatrics, British Columbia Children’s Hospital, University of British Columbia, Vancouver, British Columbia, Canada; 4 British Columbia Children's Hospital Research Institute, University of British Columbia, Vancouver, British Columbia, Canada; 5 Department Obstetrics and Gynaecology, University of British Columbia, Vancouver, British Columbia, Canada; 6 Division of Pediatric Emergency Medicine, Department of Emergency Medicine, King Abdulaziz University, Jeddah, Saudi Arabia

## Abstract

**Importance:**

Fever in the first months of life remains one of the most common pediatric problems. Urinary tract infections are the most frequent serious bacterial infections in this population. All published guidelines and quality initiatives for febrile young infants recommend lumbar puncture (LP) and cerebrospinal fluid (CSF) testing on the basis of a positive urinalysis result to exclude bacterial meningitis as a cause. For well infants older than 28 days with an abnormal urinalysis result, LP remains controversial.

**Objective:**

To assess the prevalence of bacterial meningitis among febrile infants 29 to 60 days of age with a positive urinalysis result to evaluate whether LP is routinely required.

**Data Sources:**

MEDLINE and Embase were searched for articles published from January 1, 2000, to July 25, 2018, with deliberate limitation to recent studies. Before analysis, the search was repeated (October 6, 2019) to ensure that new studies were included.

**Study Selection:**

Studies that reported on healthy, full-term, well-appearing febrile infants 29 to 60 days of age for whom patient-level data could be ascertained for urinalysis results and meningitis status were included.

**Data Extraction and Synthesis:**

Data were extracted in accordance with Preferred Reporting Items for Systematic Reviews and Meta-analyses (PRISMA) guidelines and used the Newcastle-Ottawa Scale to assess bias. Pooled prevalences and odds ratios (ORs) were estimated using random-effect models.

**Main Outcomes and Measures:**

The primary outcome was the prevalence of culture-proven bacterial meningitis among infants with positive urinalysis results. The secondary outcome was the prevalence of bacterial meningitis, defined by CSF testing or suggestive history at clinical follow-up.

**Results:**

The parent search yielded 3227 records; 48 studies were included (17 distinct data sets of 25 374 infants). The prevalence of culture-proven meningitis was 0.44% (95% CI, 0.25%-0.78%) among 2703 infants with positive urinalysis results compared with 0.50% (95% CI, 0.33%-0.76%) among 10 032 infants with negative urinalysis results (OR, 0.74; 95% CI, 0.39-1.38). The prevalence of bacterial meningitis was 0.25% (95% CI, 0.14%-0.45%) among 4737 infants with meningitis status ascertained by CSF testing or clinical follow-up and 0.28% (95% CI, 0.21%-0.36%) among 20 637 infants with positive and negative urinalysis results (OR, 0.89; 95% CI, 0.48-1.68).

**Conclusions and Relevance:**

In this systematic review and meta-analysis, the prevalence of bacterial meningitis in well-appearing febrile infants 29 to 60 days of age with positive urinalysis results ranged from 0.25% to 0.44% and was not higher than that in infants with negative urinalysis results. These results suggest that for these infants, the decision to use LP should not be guided by urinalysis results alone.

## Key Points

**Question:**

Are well-appearing febrile infants 29 to 60 days of age with positive urinalysis results at increased risk for bacterial meningitis?

**Findings:**

In a systematic review and meta-analysis of 17 distinct international data sets (25 374 infants), the pooled prevalence of bacterial meningitis among infants with positive urinalysis results ranged from 0.25% (by cerebrospinal fluid [CSF] cultures or clinical definition) to 0.44% (by CSF cultures only). Prevalence estimates were not higher compared with infants who had negative urinalysis results (0.28% by CSF cultures or clinical definition and 0.50% by CSF cultures only).

**Meaning:**

These findings suggest that, contrary to all published guidelines, invasive CSF testing in well-appearing febrile infants in the second month of life based on a positive urinalysis result alone is not supported by differential risk ratios.

## Teaser

This systematic review and meta-analysis assesses the prevalence of bacterial meningitis among febrile infants 29 to 60 days of age with a positive urinalysis result to evaluate whether lumbar puncture is routinely required.

## Introduction

Fever among infants in the first months of life remains among the most common problems in pediatric health care.^1^ These infants are at increased risk for potentially life-threatening serious bacterial infections (SBIs), specifically urinary tract infections (UTIs), bacteremia, and bacterial meningitis.^2^ Approximately 10% of febrile infants 60 days or younger have underlying UTIs,^2^ which presents a theoretical risk of hematogenous spread to the meninges. Consequently, infants with UTIs have historically been considered at increased risk for bacterial meningitis. To avoid missing 1 case of bacterial meningitis, nearly 400 infants will routinely undergo invasive cerebrospinal fluid (CSF) testing by lumbar puncture (LP), hospitalization, and broad-spectrum antibiotic therapy.^3^

Failure to detect concomitant meningitis among infants with UTIs is associated with serious sequalae. Modern urinalyses accurately predict UTIs among young infants.^4^ A presumptive diagnosis of UTI relies entirely on urinalysis results at initial evaluation before urine culture results are available. To date, all published risk-stratification strategies^5,6,7,8,9,10,11,12,13,14^ and large-scale quality improvement initiatives^15,16^ for febrile young infants include a positive urinalysis result as a high-risk feature, prompting LP, hospitalization, and empirical antibiotic treatment.

Given the changing epidemiology of SBIs^17^ and risks that decrease with infant age,^2,18^ the necessity of LP for infants older than 28 days with a presumptive UTI has been questioned for decades.^19,20^ Previous studies^21,22^ suggest that a presumptive UTI is not associated with an increased risk of bacterial meningitis among well-appearing infants older than 28 days and that urinalysis results should not alter decisions regarding CSF testing. However, given the low overall prevalence of bacterial meningitis in this age group (approximately 0.4%),^18^ no single study has been powered to determine the true risk of meningitis among well infants with a positive urinalysis result. The objectives of this study were to estimate the prevalence of bacterial meningitis among well-appearing febrile infants 29 to 60 days of age with positive urinalysis results and to compare this prevalence with that of infants with negative urinalysis results to inform whether routine LP is required.

## Methods

This study was registered prospectively in the International Prospective Register of Systematic Reviews (PROSPERO) (CRD42019122218) and followed Preferred Reporting Items for Systematic Reviews and Meta-analyses (PRISMA) reporting guideline.^23^ The British Columbia Children’s Hospital Research Ethics Board determined that ethics approval was not required.

### Search Strategy

We performed a comprehensive search of MEDLINE and Ovid Embase for articles published from January 1, 2000, to July 25, 2018. Before analysis, the search was repeated (October 6, 2019) to ensure new studies were included. Results were limited to articles published in English or French. The search was deliberately limited to studies published on or after January 1, 2000, to account for evolving clinical practice standards related to (1) changing epidemiology of SBIs attributable to widespread vaccination programs and group B *Streptococcus* prenatal screening and prophylaxis^17,18^ and (2) more uniform and stringent definitions of UTI.^24^

The search strategy was conceptualized by all study authors with the assistance of a medical librarian guided by published Medical Subject Heading terms and keywords.^25^ Broadly, the search combined the terms *fever* AND (*urinary tract infection* OR *lumbar puncture* OR *meningitis*) AND *infant* (eFigure 1 in the Supplement). Additional studies were identified through searching references of qualifying studies and systematic reviews. We included both prospective and retrospective studies with primary data. Narrative reviews, case reports, editorials, and guidelines were excluded.

### Study Selection and Definitions

Studies were eligible if they reported on previously healthy, full-term (≥37 weeks’ gestation), well-appearing (documented by unstructured physician assessment or validated observation score^26^) infants 29 to 60 days of age and evaluated for fever (documented rectal temperature of ≥38 °C). Results of urinalysis by microscopy or dipstick were eligible for inclusion.^27^ A positive urinalysis result was defined as any finding of leukocyte esterase or nitrites, a white blood cell count of 10/μL (0.01 × 10^9^/L) or higher on an uncentrifuged specimen (or ≥5/μL [0.01 × 10^9^/L] per high-power field on a centrifuged specimen), or a positive Gram stain result.^28^ Studies enrolling only infants with proven infections (viral or bacterial) or abnormal laboratory test results (eg, only infants with a positive urinalysis result) were excluded.

We contacted authors for patient-level data from studies that reported aggregate data only, those that reported UTI prevalence rather than urinalysis data specifically, and those enrolling with broader inclusion criteria. We included only studies from which we could ascertain both urinalysis results and meningitis status and only if meningitis status was determined by CSF testing or clinical follow-up when CSF was not obtained. We excluded studies in which we could not ascertain the number of infants with positive urinalysis results who underwent LP or whether meningitis status was based solely on CSF pleocytosis. To avoid double counting individual infants, publications that originated from the same source population were consolidated and analyzed as a single data set and cited by the most recent publication.

The primary outcome measure was the prevalence of definite meningitis among infants with positive urinalysis results, proven by CSF culture yielding pathogenic bacteria. The secondary outcome measure was the prevalence of meningitis among infants with positive urinalysis results, using a pragmatic clinical definition: a positive CSF culture result, bacteremia with CSF pleocytosis, or a suggestive history at clinical follow-up if CSF was not obtained.

### Identification and Data Extraction

Titles and abstracts were screened independently by 3 study investigators (V.S., F.F.M., and G.D.M.), and potentially eligible studies were evaluated for inclusion by full-text review (B.B. and F.F.M. or V.S. and G.D.M.) using standardized criteria determined a priori, with discrepancies resolved by an additional study investigator. Interreviewer agreement was tested using the Light or Cohen κ.

Data extraction was performed by 1 study investigator (B.B.) using standardized data extraction criteria, and 2 additional investigators (V.S. and G.D.M) reviewed data extraction to validate accuracy. Extracted data included study characteristics (country, methodological design, method of recruitment, study years, publication year, study definitions, and follow-up duration when applicable) and participant characteristics (age, urinalysis results, and meningitis status).

### Appraisal of Methodological Quality

Studies were assessed for methodological quality and risk of bias using the Newcastle-Ottawa Scale (NOS) checklist for nonrandomized cohort studies,^29^ as recommended by the Cochrane Collaborative.^30^ The NOS was adapted for this appraisal, and studies were considered at high risk for bias if they received less than 4 of 10 points (eFigure 2 in the Supplement).^31^ Because the data of interest were often not the primary outcomes of the included studies, items on the scale were scored on the quality of the design relative to the outcomes of this analysis rather than that of the original study objective. Two study investigators (V.S. and G.D.M.) independently appraised all data sets for methods, and discrepancies were resolved by a third (B.B.).

### Statistical Analysis

Meta-analyses were performed to assess the pooled prevalence of bacterial meningitis and to estimate odds ratios (ORs) comparing infants with positive urinalysis results and infants with negative urinalysis results. Meta-analyses were conducted using a random-effects model because substantial heterogeneity was anticipated based on differing study designs. Pooled proportions and ORs were estimated after being transformed with the logit function using generalized logistic regression models, which perform well for sparse data.^32^ Results were summarized in forest plots. To assess the robustness of pooled estimates, sensitivity analyses were planned a priori: (1) excluding studies at high risk for bias (NOS score <4), (2) analyzing only prospectively collected data sets, and (3) considering only studies with clinical follow-up of 7 days or more and those with follow-up of 30 days or more. Heterogeneity was estimated using *I*^2^ statistics and prediction intervals.^33^ A continuity correction of 0.05 was added to studies with 0 events to allow inclusion in funnel plots. Pooled proportions were assessed for publication bias by graphical inspection of funnel plots and by using the Egger test for OR estimates because there were few events per study. All analyses were performed using the meta package in R software, version 3.5.3 (R Foundation for Statistical Computing).^34^

## Results

**Figure 1.**
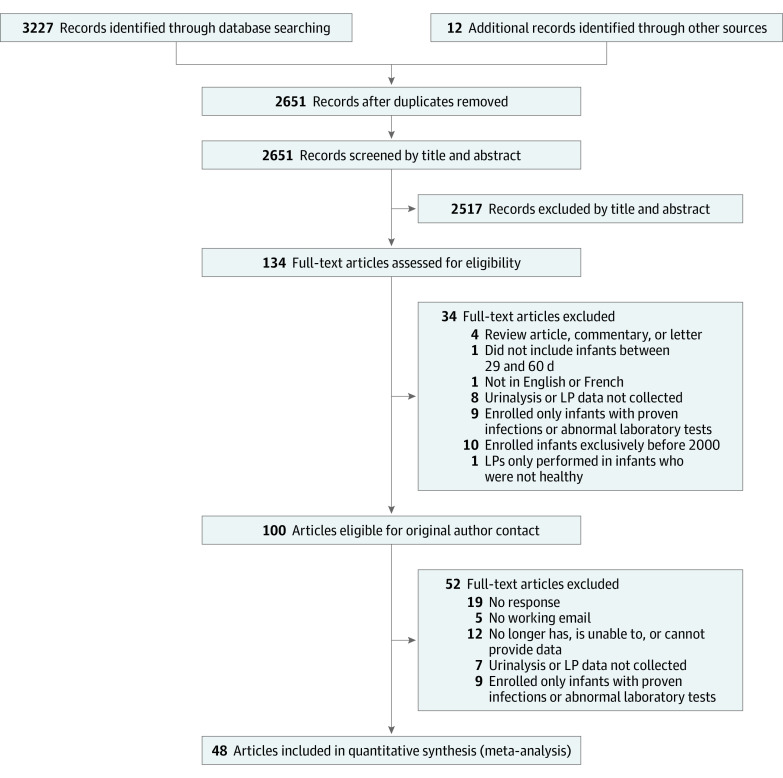
Flow Diagram of Search Results Title-abstract agreement: almost perfect for the initial 2018 search (93.1%, κ = 0.86) and the 2019 search update (92.7%, κ = 0.83). Full-text agreement: moderate for 2018 search (85.7%, κ = 0.58) and perfect for 2019 search (100.0%, κ = 1.00). LP indicates lumbar puncture.

Search of the electronic databases yielded 3227 unique publications, with an additional 12 identified by searching references of relevant studies. After removing 588 duplicates, screening by title and abstract identified 134 studies for full-text review; 34 did not meet inclusion, and 100 required author contact for patient-level data. Following contact with primary authors, 52 additional studies were excluded, 36 because authors could not be reached or were unable to provide required data (description of excluded studies in eTable 1 in the Supplement). In total, 48 individual studies^1,4,11,12,14,15,17,21,22,26,35,36,37,38,39,40,41,42,43,44,45,46,47,48,49,50,51,52,53,54,55,56,57,58,59,60,61,62,63,64,65,66,67,68,69,70,71,72^ were included for meta-analysis, with all data confirmed by the original authors for accuracy. Studies that originated from the same cohort were consolidated into a single data set for analysis. Ultimately, 17 distinct data sets were included in the analysis (Figure 1).

Descriptive characteristics of included studies are presented in Table 1, including years, study design, setting, follow-up duration, and number of eligible infants. Complete urinalysis data and meningitis status were available for 25 374 previously healthy, well-appearing full-term infants 29 to 60 days of age. All studies were published in peer-reviewed journals. Eight of 17 data sets enrolled infants in the US.^14,21,22,53,54,56,57,70^ Nine data sets were prospectively collected,^12,14,35,58,61,64,67,70,72^ and 1 study^53^ used a pre/post intervention design in which only infants from the postintervention period were analyzed prospectively. Two additional data sets were retrospective analyses^22,54^ of infants managed in the context of quality improvement initiatives. These 2 data sets were included among the sensitivity analysis of prospective studies because all relevant covariates were collected prospectively. Methodological quality of included data sets varied widely, with NOS scores ranging from 3 to 8, and 1 study^66^ at high risk for bias (eTable 2 in the Supplement).

**Table 1.** Description of Included Data Sets

Source	Study design	Enrollment years	Country	Study setting	Age of all patients enrolled	No. of infants included with urinalysis and LP/ urinalysis with or without LP	Follow-up duration	Notes
**Single-center Spanish data set**								
Bonilla et al,^35^ 2019^a^	Prospective	2003-2017	Spain	1 Pediatric ED	0-90 d	198/1141	30 d	
Gomez et al,^36^ 2019								
Mintegi et al,^37^ 2018								
Mintegi et al,^38^ 2017								
Martinez et al,^39^ 2015								
Gomez et al,^40^ 2012								
Garcia et al,^41^ 2012								
Gomez et al,^42^ 2012								
Gomez et al,^43^ 2010								
Mintegi et al,^44^ 2010								
Mintegi et al,^45^ 2009								
Benito-Fernandez et al,^46^ 2006								
**PECARN data set**								
Kuppermann et al,^14^ 2019^a^	Prospective	2008-2013	US	26 Pediatric EDs	0-60 d	2162/3110	8-14 d	Public use data set^73^ does not include infants from ongoing enrollment^74^
Ramgopal et al,^47^ 2019								
Rogers et al,^48^ 2019								
Tzimenatos et al,^4^ 2018								
Mahajan et al,^49^ 2018								
Powell et al,^50^ 2018								
Nigrovic et al,^26^ 2017								
Cruz et al,^51^ 2017								
Mahajan et al,^52^ 2016								
**REVISE data set**								
Wang et al,^22^ 2019^a^	Retrospective	2015-2017	US	124 Hospitals	7-60 d	5185/11 310	7 d	Analyzed with prospective studies, as all relevant covariates collected prospectively
Biondi et al,^15^ 2019								
**Single-study data set**								
Kasmire et al,^53^ 2019	Retrospective and prospective	2014-2017	US	1 Pediatric ED	29-60 d	87/276	30 d	Postintervention data analyzed with prospective studies
Yaeger et al,^56^ 2018	Retrospective	2014	US	1 Pediatric ED	0-90 d	32/53	7 d	
Scarfone et al,^57^ 2017	Retrospective	2007-2014	US	1 Pediatric ED	29-56 d	307/307	24 h	
Milcent et al,^61^ 2016	Prospective	2008-2011	France	15 Pediatric EDs	7-91 d	356/564	2 d	
Paquette et al,^66^ 2011	Retrospective	2001-2005	Canada	1 Pediatric ED	30-90 d	308/308	None	No follow-up data
**Kaiser Permanente data set**								
Young et al,^21^ 2018^a^	Retrospective	2007-2015	US	40 Clinics, 19 EDs, 10 pediatric inpatient units	7-90 d	583/583	30 d	No data available for infants with negative urinalysis without LP
Greenhow et al,^1^ 2016								
Greenhow et al,^17^ 2014								
**Intermountain data set**								
Blaschke et al,^54^ 2018^a^	Retrospective	2004-2016	US	21 EDs, 1 pediatric ED	1-90 d	2604/5169	3-5 d	Analyzed with prospective studies, as all relevant covariates collected prospectively
Byington et al,^55^ 2012								
**European step-by-step validation group data set**								
Gomez et al,^12^ 2016	Prospective	2014-2016	Europe	11 Pediatric EDs (8 Spain, 2 Italy, and 1 Switzerland)	0-90 d	92/622	30 d	No overlapping infants with RISeuP-SPERG data set
**RISeuP-SPERG data set**								
Gomez et al,^58^ 2016^a^	Prospective	2011-2013	Spain	19 Pediatric EDs	0-90 d	123/998	30 d	No overlapping infants with Gomez et al 2016^12^ or Bonilla et al^35^
Velasco et al,^59^ 2016								
Velasco et al,^60^ 2015								
**European group data set**								
Mintegi et al,^62^ 2014^a^	Retrospective	2008-2010	Europe	7 Pediatric EDs (5 Spain; 2 Italy)	0-90 d	39/221	None	No overlapping infants with Gomez et al 2016^12^ or Bonilla et al^35^
Bressan et al,^11^ 2012								
Gomez et al,^63^ 2012								
**Single-center Canadian data set**								
Manzano et al,^64^ 2011^a^	Prospective	2006-2007	Canada	1 Pediatric ED	1-36 mo	8/19	7 d	
Manzano et al,^65^ 2010								
**Single-center Australian data set**								
De et al,^67^ 2015^a^	Prospective	2004-2006	Australia	1 Pediatric ED	0-5 y	14/14	10-14 d	No follow-up data for those without LP
De et al,^68^ 2013								
Craig et al,^69^ 2010								
**PEM-CRC data set**								
Krief et al,^70^ 2009^a^	Prospective	1998-2001	US	8 Pediatric EDs	0-60 d	612/643	4-7 d	
Levine et al,^71^ 2004								

Abbreviations: ED, emergency department; LP, lumbar puncture; PECARN, Pediatric Emergency Care Applied Research Network; PEM-CRC, Pediatric Emergency Medicine Collaborative Research Committee; RISeuP-SPERG, Spanish Pediatric Emergency Research Group of the Spanish Society of Pediatric Emergencies; REVISE, Reducing Variability in the Infant Sepsis Evaluation.

^a^ Representative study for full consolidated data set.

Among included data sets, meningitis status was determined by CSF testing for 12 735 infants and by CSF testing or clinical follow-up for 25 374 infants. For both the primary and secondary outcomes, the unweighted proportion of infants with bacterial meningitis was higher among infants with negative urinalysis results in 9 data sets,^14,21,22,35,53,54,58,66,70^ higher among infants with positive urinalysis results in a single data set,^61^ and 0 in both groups in 7 data sets.^12,56,57,62,64,67,72^ The 7 data sets reporting 0 cases of meningitis in both groups did not contribute to the pooled OR, and several data sets had estimated ORs greater than 1 despite 0 events in the group with positive urinalysis results because of imbalanced sample sizes.

**Figure 2.**
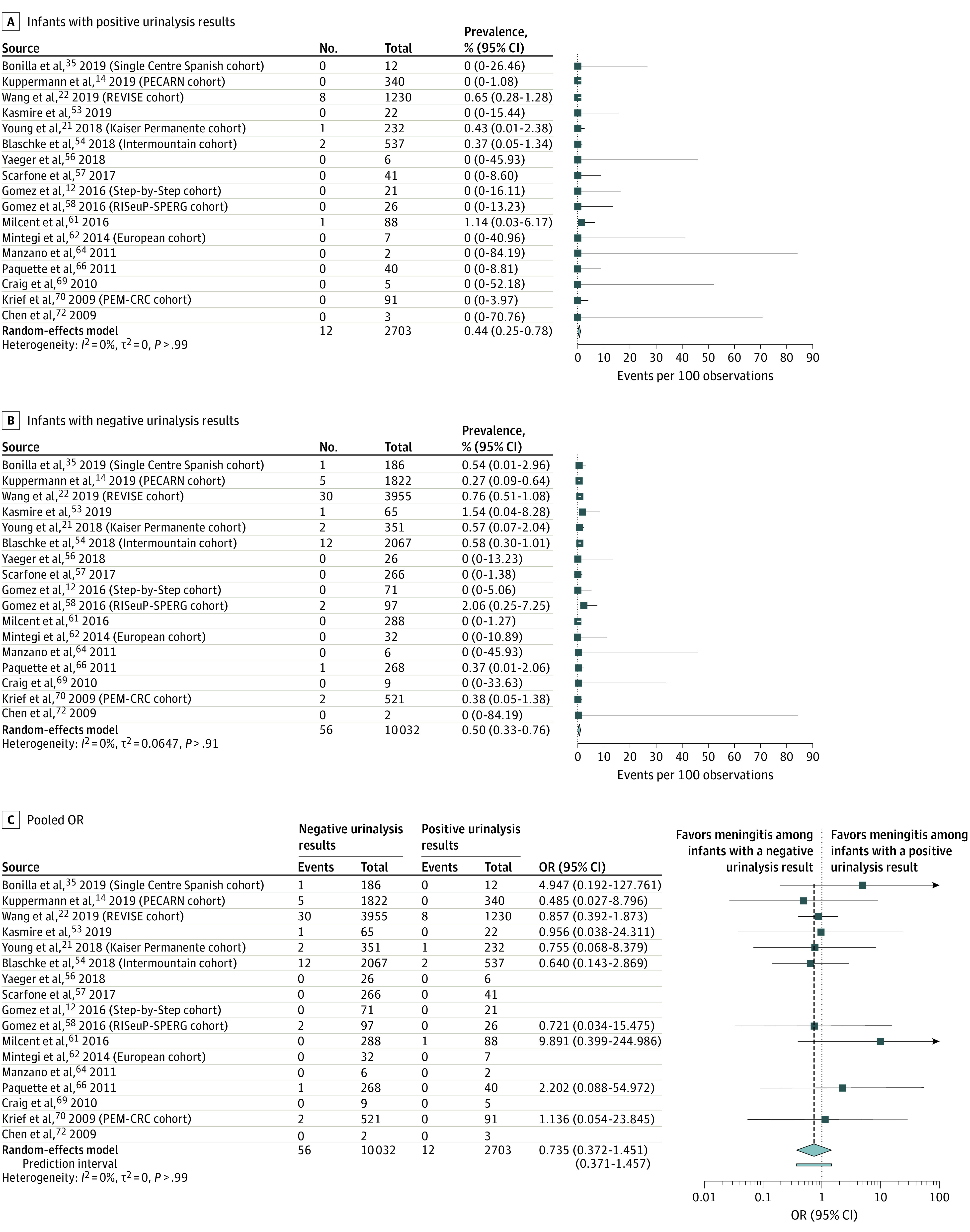
Forest Plots of Pooled Prevalence of Culture-Proven Bacterial Meningitis For the pooled odds ratio (OR) analysis (C), the arrow indicates that the upper confidence limit falls beyond the x-axis; diamond, the overall estimate from the meta-analysis and its confidence interval, with the center of the diamond representing the pooled estimate; and the bar below the diamond, the prediction interval.

For the primary outcome measure (Figure 2), there were 12 cases of culture-proven meningitis among 2703 infants with positive urinalysis results (95% CI, 0.25%-0.78%) and 56 cases among 10 032 infants with negative urinalysis results (OR, 0.74; 95% CI, 0.39-1.38). The pooled prevalence of bacterial meningitis was 0.44% (95% CI, 0.25%-0.78%; *I*^2^ = 0%) (Figure 2A) among infants with positive urinalysis results and 0.50% (95% CI, 0.33%-0.76%; *I*^2^ = 14%) (Figure 2B) among infants with negative urinalysis results (pooled OR, 0.74; 95% CI, 0.39%-1.38%; *I*^2^ = 0%) (Figure 2C).

**Figure 3.**
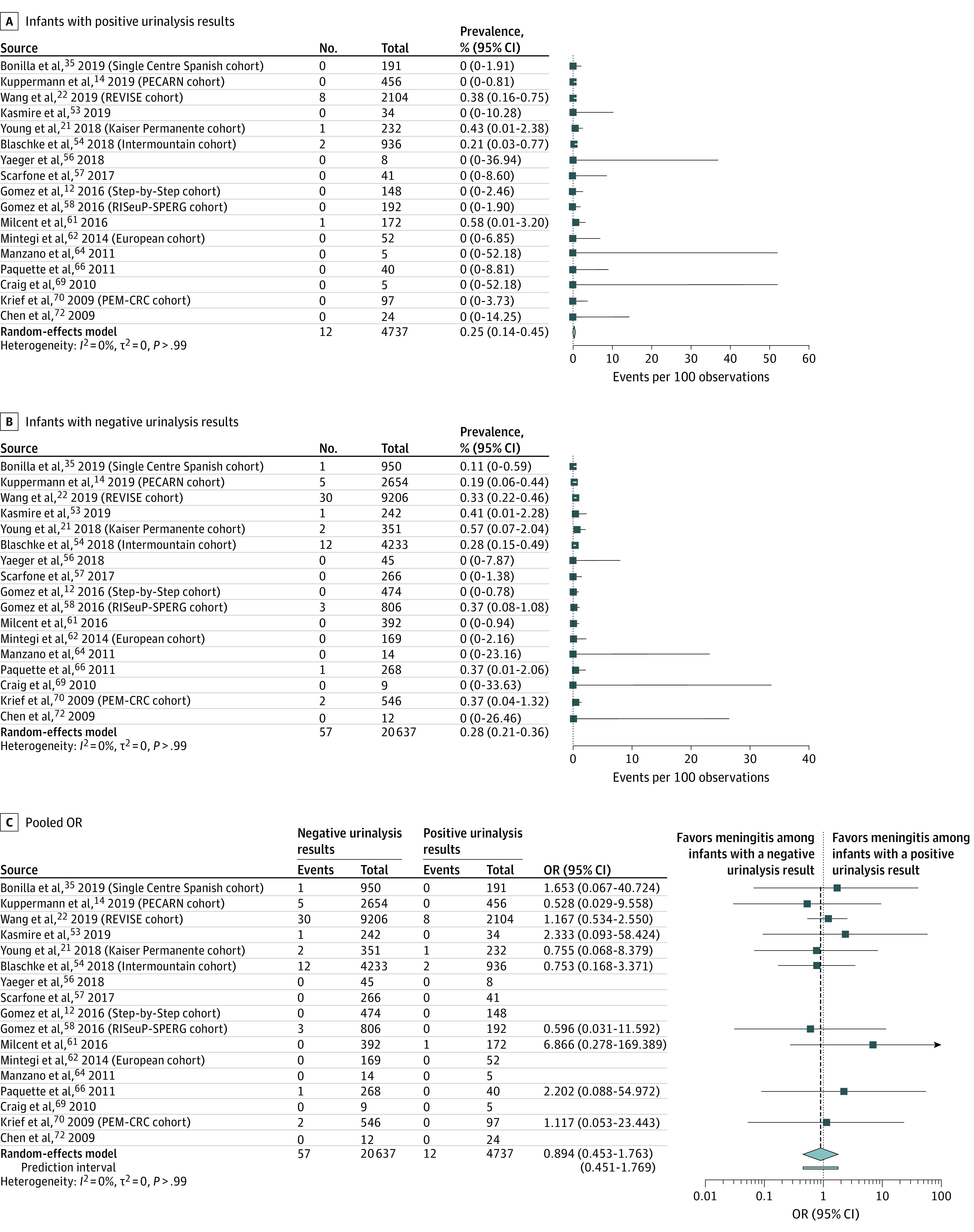
Forest Plots of Pooled Prevalence of Bacterial Meningitis Determined by Cerebrospinal Fluid Testing or Clinical Follow-up For the pooled odds ratio (OR) analysis (C), the arrow indicates that the upper confidence limit falls beyond the x-axis; diamond, the overall estimate from the meta-analysis and its confidence interval, with the center of the diamond representing the pooled estimate; and the bar below the diamond, the prediction interval.

For the secondary outcome measure of bacterial meningitis status determined by CSF testing or clinical follow-up (Figure 3), there were 12 cases among 4737 infants with positive urinalysis results and 57 cases among 20 637 infants with negative urinalysis results. The pooled prevalence was 0.25% (95% CI, 0.14%-0.45%; *I*^2^ = 0%) (Figure 3A) among infants with positive urinalysis results and 0.28% (95% CI, 0.21%-0.36%; *I*^2^ = 0%) (Figure 3B) among infants with negative urinalysis results (pooled OR, 0.89; 95% CI, 0.48%-1.68%; *I*^2^ = 0%) (Figure 3C).

Funnel plots for both outcome measures demonstrated symmetrical distributions around all pooled estimates, graphically not suggestive of publication bias (eFigure 3 in the Supplement). The Egger test for the primary and secondary outcome OR estimates were similarly not suggestive of publication bias.

We conducted sensitivity analyses (1) excluding studies at high risk for bias, (2) analyzing only prospectively collected data, and (3) considering only studies with clinical follow-up of 7 days or more and 30 days or more (eFigures 4, 5, 6, and 7 in the Supplement). The estimates for data sets with 30 days or more of follow-up (eFigure 7 in the Supplement) were most different from the full analysis because of inclusion of the fewest data sets (n = 5) but produced the lowest ORs. Overall, results of sensitivity analyses did not differ significantly, lending confidence to findings of the primary analyses (Table 2).

**Table 2.** Summary of Sensitivity Analyses

Data set	Primary outcome measure			Secondary outcome measure		
	Pooled prevalence, % (95% CI)		Pooled OR (95% CI)	Pooled prevalence, % (95% CI)		Pooled OR (95% CI)
	Positive urinalysis results	Negative urinalysis results		Positive urinalysis results	Negative urinalysis results	
All data sets (N = 17)	0.44 (0.25-0.78)	0.50 (0.33-0.76)	0.74 (0.39-1.38)	0.25 (0.14-0.45)	0.28 (0.21-0.36)	0.89 (0.48- 1.68)
Excluding data sets at high risk for bias (n = 16)	0.45 (0.26-0.79)	0.51 (0.33-0.77)	0.74 (0.40-1.39)	0.26 (0.15-0.45)	0.27 (0.21-0.36)	0.90 (0.48-1.70)
Prospective data sets only (n = 12)^a^	0.46 (0.26-0.84)	0.54 (0.36-0.82)	0.73 (0.38-1.40)	0.25 (0.14-0.46)	0.28 (0.21-0.36)	0.92 (0.48-1.75)
Data sets with ≥7 d of follow-up (n = 10)	0.47 (0.25-0.91)	0.58 (0.36-0.93)	0.71 (0.34-1.47)	0.27 (0.14-0.51)	0.28 (0.21-0.39)	0.89 (0.43-1.85)
Data sets with ≥30 d of follow-up (n = 5)	0.32 (0.04-2.23)	0.78 (0.35-1.72)	0.42 (0.05-3.62)	0.13 (0.02-0.89)	0.25 (0.12-0.52)	0.39 (0.05- 3.32)

Abbreviation: OR, odds ratio.

^a^ Nine data sets were prospectively collected, 1 data set had prospective postintervention data, and 2 retrospective data sets were analyzed with prospective studies because of prospective collection of all relevant covariates.

## Discussion

This systematic review and meta-analysis is the largest and most comprehensive study, to our knowledge, to evaluate the risk among well-appearing febrile infants older than 28 days with a positive urinalysis result. Accurate prevalence estimates are essential for practitioners to quantify the risk of concomitant bacterial meningitis and to inform clinical decision-making. The present analysis combined data from 17 unique data sets with more than 25 000 infants from geographically diverse populations. The results suggest that infants with a positive urinalysis result are at no higher risk for bacterial meningitis than infants with a negative urinalysis result. This finding is contrary to the dogma held for nearly 30 years.^6^ Historically, all risk-stratification criteria, including very recently derived clinical decision rules,^5,6,7,8,9,10,11,12,13,14^ categorize a positive urinalysis result as a high-risk feature, prompting invasive testing, broad-spectrum antibiotic exposure, and hospitalization. Although this practice has been controversial for decades, the very low overall prevalence of bacterial meningitis has meant that no single study could reliably answer this clinical question.^20,21,22,31,66^ This large meta-analysis provides compelling evidence that decisions regarding LP for this subgroup of infants should not be guided by urinalysis results alone.

The evaluation of febrile young infants is invasive, anxiety-provoking for parents, and associated with iatrogenic risk and significant system-wide resource use.^75^ Since 2016, improved care for febrile young infants has become the largest US-wide quality initiative ever endorsed by the American Academy of Pediatrics, including 124 independent hospitals across 38 US states.^15^ Widely disseminated clinical pathways and electronic decision-support tools have been developed, which classify infants 29 to 60 days of age with a positive urinalysis result at increased risk for bacterial meningitis and recommend LP; conversely, infants with a negative urinalysis result are classified as low risk, and LP is not required.^16^ Findings from this analysis are in contrast to these recommendations.

### Strengths and Limitations

One strength of the current analysis is the calculation of pooled ORs rather than only prevalence estimates,^31^ which must be compared with historical controls.^18^ This analysis allows a direct comparison of prevalences and an estimation of odds among infants with positive and negative urinalysis results within included studies. Of note, the pooled ORs for the primary and secondary outcomes were below 1, supporting the conclusion that infants with positive urinalysis results are not at higher risk for bacterial meningitis. In fact, the prevalence of meningitis was higher among infants with positive urinalysis results in just a single medium-sized data set.^61^ Moreover, another large data set^74^ has since enrolled many additional infants with an even lower relative risk among those with positive urinalysis results than was available at the time of analysis. Furthermore, 3 data sets reported 0 cases of meningitis in the urinalysis-positive group and 1 case or more in the urinalysis-negative group but generated ORs greater than 1.^35,66,70^ Paradoxical ORs such as these are possible when there is an imbalance in sample sizes, and the smaller sample has 0 events. Inclusion of these studies is known to bias pooled ORs toward the null.^76^ Taken together, it is likely that pooled OR point estimates reported are, if anything, an overestimate.

A novel contribution of this analysis was the purposeful exclusion of studies before the year 2000 to account for changing SBI epidemiology.^17,18^ The current analysis differs importantly from a recent small meta-analysis,^31^ which consisted primarily of retrospective studies using a culture-proven UTI case definition. Similar to estimates reported here, Nugent et al^31^ reported 11 cases of bacterial meningitis among 3868 infants 29 to 90 days of age with an abnormal urinalysis result or culture-confirmed UTI who underwent LP (pooled prevalence, 0.25%). However, their analysis did not estimate the prevalence among infants with negative urinalysis results; thus, no direct comparison could be made or OR calculated. In addition, results of this analysis were driven largely by a single study^77^ that contributed 1609 infants selected on the basis of a culture-confirmed UTI not urinalysis results. Of importance, clinical decisions about LP and hospitalization rely on initial urinalysis results not urine culture results. A urinalysis is highly sensitive (0.94; 95% CI, 0.91-0.97) and specific (0.91; 95% CI, 0.90-0.91) for UTIs in febrile young infants.^4^ A urinalysis is also the most universally used diagnostic test for risk stratification,^2,48^ and an abnormal urinalysis result is among the most frequent reasons that infants do not meet low-risk criteria.^57^

There are several additional strengths of the current analysis. The study population does not include infants from studies that selected infants on the basis of a clear focus of infection and thus addresses the most common and challenging clinical conundrum when evaluating well-appearing febrile young infants. Authors were contacted to obtain patient-level data and to accurately consolidate overlapping studies to prevent repeat counting of individual infants. Sensitivity analyses were selected a priori and increase the confidence in the results of the main analysis. Measures were taken to assess the possibility of publication bias, which does not appear to have influenced the results to any significant degree.

This analysis also has limitations. It is possible that the pooled prevalence of meningitis among infants with negative urinalysis results reported is falsely elevated because infants with a normal urinalysis result who did not undergo CSF testing would not be included in the denominator. However, studies with clinical follow-up of at least 7 and 30 days would capture these infants, and sensitivity analyses reveal OR point estimates that are still not higher among infants with positive urinalysis results. The secondary outcome used a pragmatic clinical definition of meningitis, and neither a threshold for CSF pleocytosis^78,79,80^ nor a definition of history suggestive of meningitis at follow-up was prespecified; rather, the outcomes were reported as classified by the primary study authors. Fifty-two studies were excluded (several with overlapping data sets), including 36 for which patient-level data were not available. Bias introduced by their exclusion is theoretically possible; however, it is unlikely that these infants were systematically different from those analyzed. In all studies, the decision to perform an LP was at the discretion of the treating physician; however, the sensitivity analysis limited to studies with follow-up of 30 days or more with the lowest pooled ORs mitigates the risk of missing cases of bacterial meningitis among infants without CSF testing or a short clinical follow-up. Although every attempt has been made to not double count infants, the possibility cannot be completely excluded given that several included studies were large national or multinational studies, although most data sets did not overlap temporally or geographically. Only studies published in English or French were included, and most were conducted in emergency departments; therefore, estimates may not be generalizable to other settings (ie, ambulatory clinics or unrepresented countries). In addition, pooled prevalence estimates are associated with the urinalysis status in isolation, and the risk when other diagnostic biomarkers are also within normal limits was not assessed (ie, C-reactive protein and procalcitonin). Studies with 0 events provide challenges in estimation with traditional meta-analytic methods; as such, generalized linear mixed-effects models were used to compensate. High heterogeneity across studies was expected, and qualitatively this was true based on study methods. Despite this, for some outcomes, the *I*^2^ statistics could not be accurately estimated because of the small (and 0) event rates in many studies.^76^ For these estimates, the prediction intervals must be relied on to provide a relevant alternative measure of the heterogeneity and are reported for all analyses.

## Conclusions

Invasive CSF testing, hospitalization, and empirical antibiotic treatment of well-appearing febrile infants older than 28 days with a positive urinalysis result have been predicated for decades on the assumption of an increased risk of bacterial meningitis. Despite fever in young infants being a common clinical problem, no single study to date has been adequately large to reliably determine the true relative risk among infants with positive urinalysis results. Findings from this large meta-analysis suggest that well-appearing febrile infants 29 to 60 days of age with a positive urinalysis result are not at an elevated risk for bacterial meningitis compared with infants with negative urinalysis results. Overall, these results suggest that the rate of concomitant bacterial meningitis in this population is low, and LP should not be undertaken on the basis of a positive urinalysis result alone.

## Notes

Supplement. **eFigure 1.** Search Strategies for MEDLINE and Embase **eFigure 2.** Newcastle-Ottawa Scale Critical Appraisal Tool **eFigure 3.** Funnel Plots for Primary Outcomes (A-C; Pooled Prevalence of Bacterial Meningitis Among Urinalysis-Positive Infants, Urinalysis-Negative Infants and Pooled Odds Ratio) and Secondary Outcomes (D-F; Pooled Prevalence of Bacterial Meningitis Among Urinalysis-Positive Infants, Urinalysis-Negative Infants and Pooled Odds Ratio) **eFigure 4.** Forest Plots of Sensitivity Analysis; Pooled Prevalence of Bacterial Meningitis Among Urinalysis-Positive Infants, Urinalysis-Negative Infants and Pooled Odds Ratio for Primary (A-C) and Secondary (D-F) Outcome Measures, Excluding Studies at High Risk of Bias **eFigure 5.** Forest Plots of Sensitivity Analysis; Pooled Prevalence of Bacterial Meningitis Among Urinalysis-Positive Infants, Urinalysis-Negative Infants and Pooled Odds Ratio for Primary (A-C) and Secondary (D-F) Outcome Measures, Including Prospective Studies Only **eFigure 6.** Forest Plots of Sensitivity Analysis; Pooled Prevalence of Bacterial Meningitis Among Urinalysis-Positive Infants, Urinalysis-Negative Infants and Pooled Odds Ratio for Primary (A-C) and Secondary (D-F) Outcome Measures, Including Studies With ≥7 Days Clinical Follow-up **eFigure 7.** Forest Plots of Sensitivity Analysis; Pooled Prevalence of Bacterial Meningitis Among Urinalysis-Positive Infants, Urinalysis-Negative Infants and Pooled Odds Ratio for Primary (A-C) and Secondary (D-F) Outcome Measures, Including Studies With ≥30 Days Clinical Follow-up **eTable 1.** Description of Studies Excluded After Primary Author Contact **eTable 2.** Results of Critical Appraisal Checklist for Studies Included in Meta-analysis eReferences
