# Supplementary material for: Prevalence of Bacterial Meningitis Among Febrile Infants Aged 29-60 Days With Positive Urinalysis Results: A Systematic Review and Meta-analysis
Source: JAMA Netw Open. 2021 May 12;4(5):e214544. doi: 10.1001/jamanetworkopen.2021.4544 (PMC8116985; doi:10.1001/jamanetworkopen.2021.4544)
Supplement: Supplement. — eFigure 1. Search Strategies for MEDLINE and Embase eFigure 2. Newcastle-Ottawa Scale Critical Appraisal Tool eFigure 3. Funnel Plots for Primary Outcomes (A-C; Pooled Prevalence of Bacterial Meningitis Among Urinalysis-Positive Infants, Urinalysis-Negative Infants and Pooled Odds Ratio) and Secondary Outcomes (D-F; Pooled Prevalence of Bacterial Meningitis Among Urinalysis-Positive Infants, Urinalysis-Negative Infants and Pooled Odds Ratio) eFigure 4. Forest Plots of Sensitivity Analysis; Pooled Prevalence of Bacterial Meningitis Among Urinalysis-Positive Infants, Urinalysis-Negative Infants and Pooled Odds Ratio for Primary (A-C) and Secondary (D-F) Outcome Measures, Excluding Studies at High Risk of Bias eFigure 5. Forest Plots of Sensitivity Analysis; Pooled Prevalence of Bacterial Meningitis Among Urinalysis-Positive Infants, Urinalysis-Negative Infants and Pooled Odds Ratio for Primary (A-C) and Secondary (D-F) Outcome Measures, Including Prospective Studies Only eFigure 6. Forest Plots of Sensitivity Analysis; Pooled Prevalence of Bacterial Meningitis Among Urinalysis-Positive Infants, Urinalysis-Negative Infants and Pooled Odds Ratio for Primary (A-C) and Secondary (D-F) Outcome Measures, Including Studies With ≥7 Days Clinical Follow-up eFigure 7. Forest Plots of Sensitivity Analysis; Pooled Prevalence of Bacterial Meningitis Among Urinalysis-Positive Infants, Urinalysis-Negative Infants and Pooled Odds Ratio for Primary (A-C) and Secondary (D-F) Outcome Measures, Including Studies With ≥30 Days Clinical Follow-up eTable 1. Description of Studies Excluded After Primary Author Contact eTable 2. Results of Critical Appraisal Checklist for Studies Included in Meta-analysis eReferences [file jamanetwopen-e214544-s001.pdf]

Supplementary Online Content

Burstein B, Sabhaney V, Bone JN, Doan Q, Mansouri FF, Meckler GD. Prevalence of bacterial meningitis among febrile infants aged 29-60 days with positive urinalysis results: a systematic review and meta-analysis. *JAMA Netw Open*. 2021;4(5):e214544. doi:10.1001/jamanetworkopen.2021.4544

eFigure 1. Search Strategies for MEDLINE (A) and Embase (B)

eFigure 2. Newcastle-Ottawa Scale Critical Appraisal Tool

eFigure 3. Funnel Plots for Primary Outcomes (A-C; Pooled Prevalence of Bacterial Meningitis Among Urinalysis-Positive Infants, Urinalysis-Negative Infants and Pooled Odds Ratio) and Secondary Outcomes (D-F; Pooled Prevalence of Bacterial Meningitis Among Urinalysis-Positive Infants, Urinalysis-Negative Infants and Pooled Odds Ratio)

eFigure 4. Forest Plots of Sensitivity Analysis; Pooled Prevalence of Bacterial Meningitis Among Urinalysis-Positive Infants, Urinalysis-Negative Infants and Pooled Odds Ratio for Primary (A-C) and Secondary (D-F) Outcome Measures, Excluding Studies at High Risk of Bias

eFigure 5. Forest Plots of Sensitivity Analysis; Pooled Prevalence of Bacterial Meningitis Among Urinalysis-Positive Infants, Urinalysis-Negative Infants and Pooled Odds Ratio for Primary (A-C) and Secondary (D-F) Outcome Measures, Including Prospective Studies Only

eFigure 6. Forest Plots of Sensitivity Analysis; Pooled Prevalence of Bacterial Meningitis Among Urinalysis-Positive Infants, Urinalysis-Negative Infants and Pooled Odds Ratio for Primary (A-C) and Secondary (D-F) Outcome Measures, Including Studies With ≥7 Days Clinical Follow-up

eFigure 7. Forest Plots of Sensitivity Analysis; Pooled Prevalence of Bacterial Meningitis Among Urinalysis-Positive Infants, Urinalysis-Negative Infants and Pooled Odds Ratio for Primary (A-C) and Secondary (D-F) Outcome Measures, Including Studies With ≥30 Days Clinical Follow-up

eTable 1. Description of Studies Excluded After Primary Author Contact

eTable 2. Results of Critical Appraisal Checklist for Studies Included in Meta-Analysis

eReferences

This supplementary material has been provided by the authors to give readers additional information about their work.

eFigure 1. Search strategies for MEDLINE (A) and Embase (B)

A) MEDLINE

Database: Ovid MEDLINE(R) Epub Ahead of Print, In-Process & Other Non-Indexed Citations, Ovid MEDLINE(R) Daily and Ovid MEDLINE(R) <1946 to Present>
Search Strategy:
----
1 exp Fever/
2 exp Fever of Unknown Origin/
3 fever.mp.
4 febrile.mp.
5 (pyrexia or pyrexias).mp.
6 or/1-5 [Fever]
7 exp urinary tract infections/
8 (urinary tract adj3 infection?).mp.
9 UTI.mp.
10 exp bacteriuria/
11 (bacteriuria or bacteriurias).mp.
12 exp pyuria/
13 (pyuria or pyurias).mp.
14 exp Pyelonephritis/
15 exp cystitis/
16 exp pyelocystitis/
17 (Pyelonephritis or cystitis or pyelocystitis).mp.
18 or/7-17 [UTI]
19 6 and 18
20 exp Spinal Puncture/
21 ((spinal or lumbar) adj3 (tap? or puncture?)).mp.
22 Cerebrospinal Fluid/
23 Cerebrospinal Fluid.mp.
24 CSF.mp.
25 or/20-24 [Spinal tap]
26 6 and 25
27 exp meningitis/
28 exp arachnoiditis/
29 exp meningitis, bacterial/ or exp meningitis, escherichia coli/ or exp meningitis, haemophilus/ or exp meningitis, listeria/ or exp meningitis, meningococcal/ or exp waterhouse-friderichsen syndrome/
30 exp meningitis, pneumococcal/
31 exp tuberculosis, meningeal/
32 meningit$.mp.
33 pachymeningitides.mp.
34 pachymeningitis.mp.
35 listeria cerebriti$.mp.
36 listeria meningoencephaliti$.mp.
37 listeria monocytogenes meningiti$.mp.

© 2021 Burstein B et al. *JAMA Network Open.*

38 waterhouse-friederichsen syndrome.mp.
39 exp central nervous system infections/ or exp brain abscess/ or exp central nervous system bacterial infections/ or exp encephalomyelitis/ or exp epidural abscess/ or exp infectious encephalitis/ or exp meningoencephalitis/ or exp myelitis/ or exp perimeningeal infections/
40 (central nervous system adj2 infection?).mp.
41 or/27-40 [Meningitis]
42 6 and 41
43 exp infant/
44 exp infant, newborn/
45 exp infant, low birth weight/
46 exp infant, small for gestational age/
47 (paediatric? or pediatric?).mp.
48 (infant? or infancy or infantile or newborn? or neonat$).mp.
49 or/43-48
50 6 and (18 or 25 or 41) and 49
51 animals/ not (human/ and animals/)
52 50 not 51
53 comment/ or editorial/ or letter/ or news/
54 52 not 53
55 limit 54 to (english or french)
56 54 not 55 [Non-English]
57 limit 55 to systematic reviews
58 limit 55 to "review articles"
59 limit 55 to "reviews (best balance of sensitivity and specificity)"
60 or/57-59 [Reviews]
61 55 not 60 [Remaining References]
62 limit 61 to yr="2000 -Current"

B) Embase
Database: Embase <1996 to 2019 Week 40>
Search Strategy:
----
1 *fever/
2 *pyrexia idiopathica/
3 fever.ti,ab,kw.
4 febrile.ti,ab,kw.
5 (pyrexia or pyrexias).ti,ab,kw.
6 or/1-5
7 exp *urinary tract infection/
8 (urinary tract adj3 infection?).ti,ab,kw.
9 UTI.ti,ab,kw.
10 *bacteriuria/
11 (bacteriuria or bacteriurias).ti,ab,kw.
12 *pyuria/
13 (pyuria or pyurias).ti,ab,kw.
14 exp *Pyelonephritis/

15 exp *cystitis/
16 (Pyelonephritis or cystitis or pyelocystitis).ti,ab,kw.
17 or/7-16
18 6 and 17
19 *lumbar puncture/
20 ((spinal or lumbar) adj3 (tap? or puncture?)).ti,ab,kw.
21 *Cerebrospinal Fluid/
22 Cerebrospinal Fluid.ti,ab,kw.
23 CSF.ti,ab,kw.
24 or/19-23
25 6 and 24
26 *meningitis/
27 *arachnoiditis/
28 *bacterial meningitis/ or *Haemophilus meningitis/ or *Listeria meningitis/ or exp *meningococcosis/
29 exp *meningitis, pneumococcal/
30 exp *tuberculosis, meningeal/
31 meningit$.ti,ab,kw.
32 pachymeningitides.ti,ab,kw.
33 pachymeningitis.ti,ab,kw.
34 listeria cerebriti$.ti,ab,kw.
35 listeria meningoencephaliti$.ti,ab,kw.
36 listeria monocytogenes meningiti$.ti,ab,kw.
37 waterhouse-friederichsen syndrome.ti,ab,kw.
38 exp *central nervous system infections/ or exp *brain abscess/ or exp *central nervous system bacterial infections/ or exp *encephalomyelitis/ or exp *epidural abscess/ or exp *infectious encephalitis/ or exp *meningoencephalitis/ or exp *myelitis/ or exp *perimeningeal infections/
39 (central nervous system adj2 infection?).ti,ab,kw.
40 or/26-39
41 6 and 40
42 exp *infant/
43 exp *low birth weight/
44 (paediatric? or pediatric?).ti,ab,kw.
45 (infant? or infancy or infantile or newborn? or neonat$).ti,ab,kw.
46 or/42-45
47 6 and (17 or 24 or 40) and 46
48 (exp animal/ or nonhuman/) not exp human/
49 47 not 48
50 editorial/ or letter/ or exp review/
51 49 not 50
52 limit 51 to (english or french)
53 51 not 52 [Non-English]
54 limit 52 to yr="2000 -Current"
55 remove duplicates from 54

eFigure 2. Newcastle-Ottawa Scale critical appraisal tool

1) Was the sample representative of the target population?
1 star: Sample includes well-appearing, previously healthy term infants aged 29-60 days with urinalysis performed evaluated for fever documented at home or in health care setting
0 stars: Study includes infants aged 29-60 days with urinalysis performed and not all patients had a documented fever (example; included tactile or subjectively reported)
2) Were study participants recruited in an appropriate way?
1 star: Study patients were identified prospectively
0 stars: Study patients were identified retrospectively
3) Sample size*
2 stars: >5106 infants aged 29-90 days with urinalysis underwent CSF testing
1 star: >1276 infants aged 29-60 days with urinalysis underwent CSF testing
0 stars: <1277 infants aged 29-60 days with urinalysis underwent CSF testing
4) Quality of descriptive statistics reporting
1 star: Reports descriptive statistics about the population (minimum age, prematurity, comorbidity, appearance, focal infection)
0 stars: Any of the descriptive statistics about the population missing
5) Selection bias: Is the data analysis conducted with sufficient coverage of the identified sample?
2 stars: All infants aged 29-60 days with urinalysis performed underwent CSF testing
1 star: ≥50% of infants aged 29-60 days with urinalysis performed underwent CSF testing
0 stars: <50% of infants aged 20-60 days with urinalysis performed underwent CSF testing, 0 points if retrospective review of only infants who had both urinalysis and CSF collected but no mention of infants with urinalysis who did not undergo CSF testing
6) Ascertainment of urinalysis results
1 star: Reports diagnostic criteria for abnormal urinalysis and requires catheterization urine or supra-pubic aspiration
0 stars: Fails to report diagnostic criteria for abnormal urinalysis or includes clean-catch or bag cultures
7) Assessment of outcome
1 star: Reports diagnostic criteria for bacterial meningitis and excludes bacterial cultures that are likely to be contaminants
0 stars: Fails to report diagnostic criteria for bacterial meningitis or does not exclude likely contaminants
8) Adequacy of follow-up
1 star: CSF culture results or clinical follow-up was available for ≥90% of patients
0 stars: >10% of patients had incomplete CSF culture data or clinical follow-up

*Sample Size[1]
Goal *n* = 5107 for bacterial meningitis with estimated prevalence of 0.3% +/- 0.15%
Goal *n* = 1277 for bacterial meningitis with estimated prevalence of 0.3% +/- 0.3%

eFigure 3. Funnel plots for primary outcomes (A-C; pooled prevalence of bacterial meningitis among Urinalysis-positive infants, Urinalysis-negative infants and pooled odds ratio) and secondary outcomes (D-F; pooled prevalence of bacterial meningitis among Urinalysis-positive infants, Urinalysis-negative infants and pooled odds ratio).

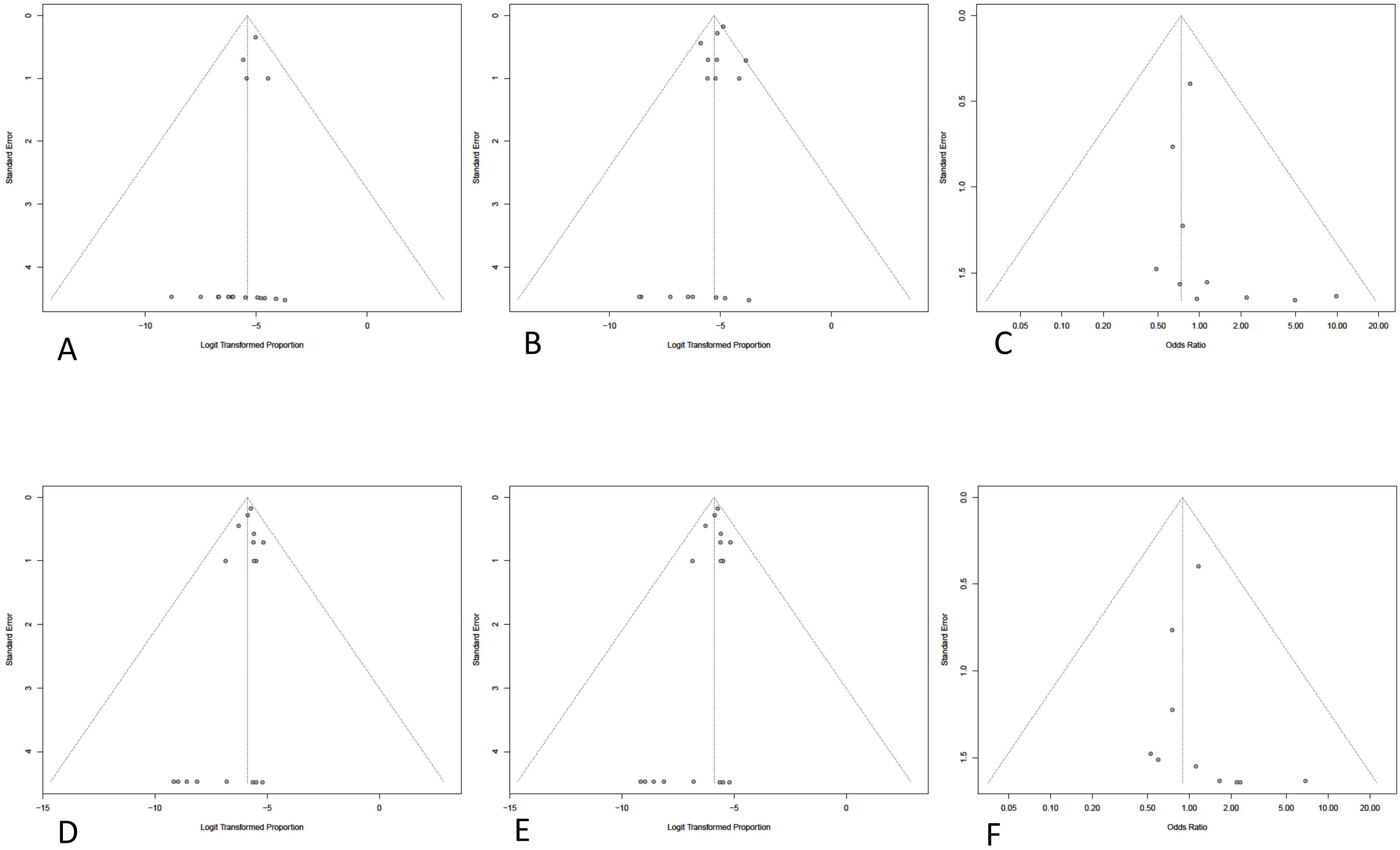

eFigure 4. Forest plots of sensitivity analysis; pooled prevalence of bacterial meningitis among Urinalysis-positive infants, Urinalysis-negative infants and pooled odds ratio for primary (A-C) and secondary (D-F) outcome measures, excluding studies at high risk of bias.

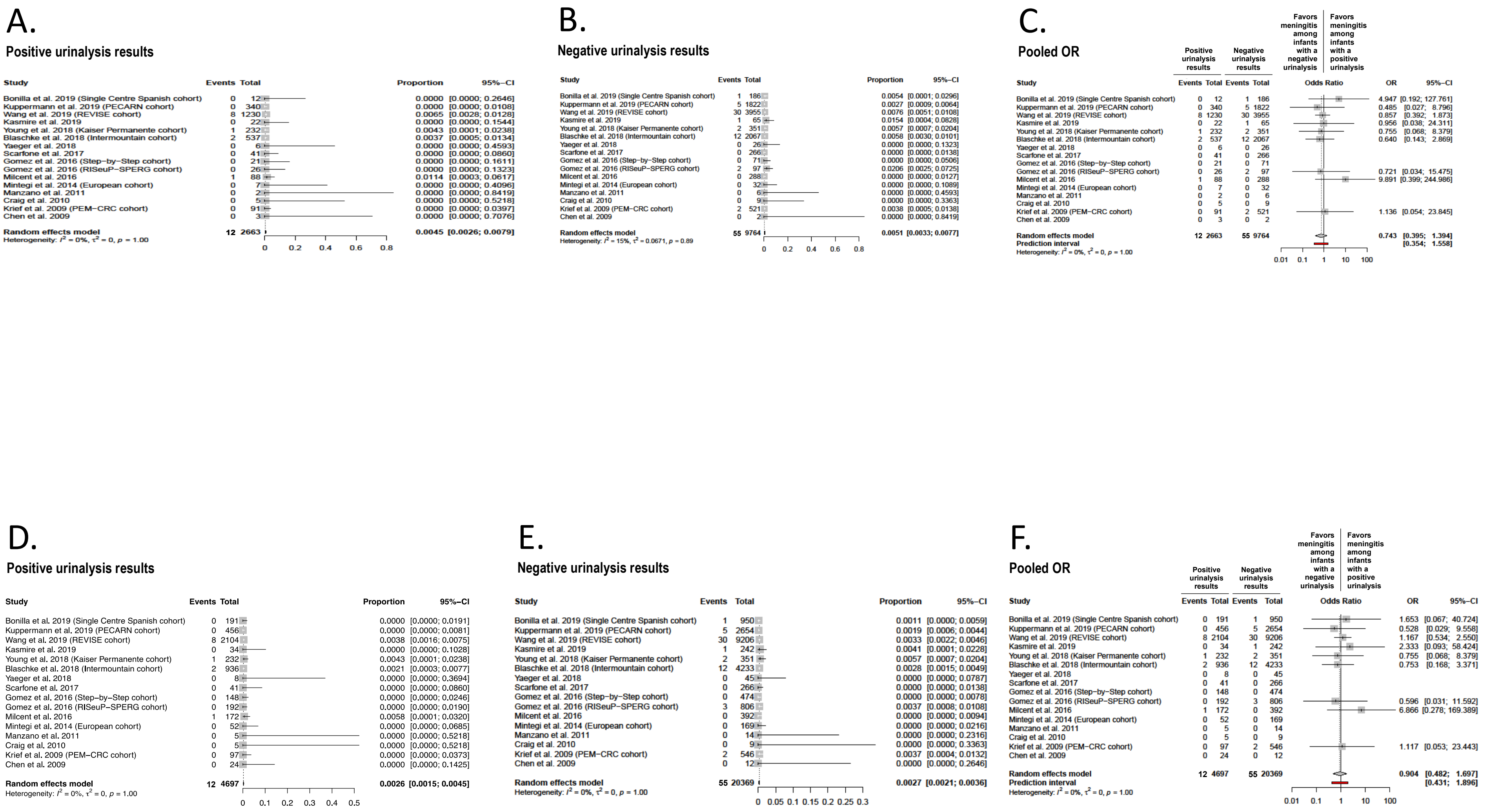

eFigure 5. Forest plots of sensitivity analysis; pooled prevalence of bacterial meningitis among urinalysis-positive infants, urinalysis-negative infants and pooled odds ratio for primary (A-C) and secondary (D-F) outcome measures, including prospective studies only.
*Analyzed with prospective studies, as all relevant covariates collected prospectively

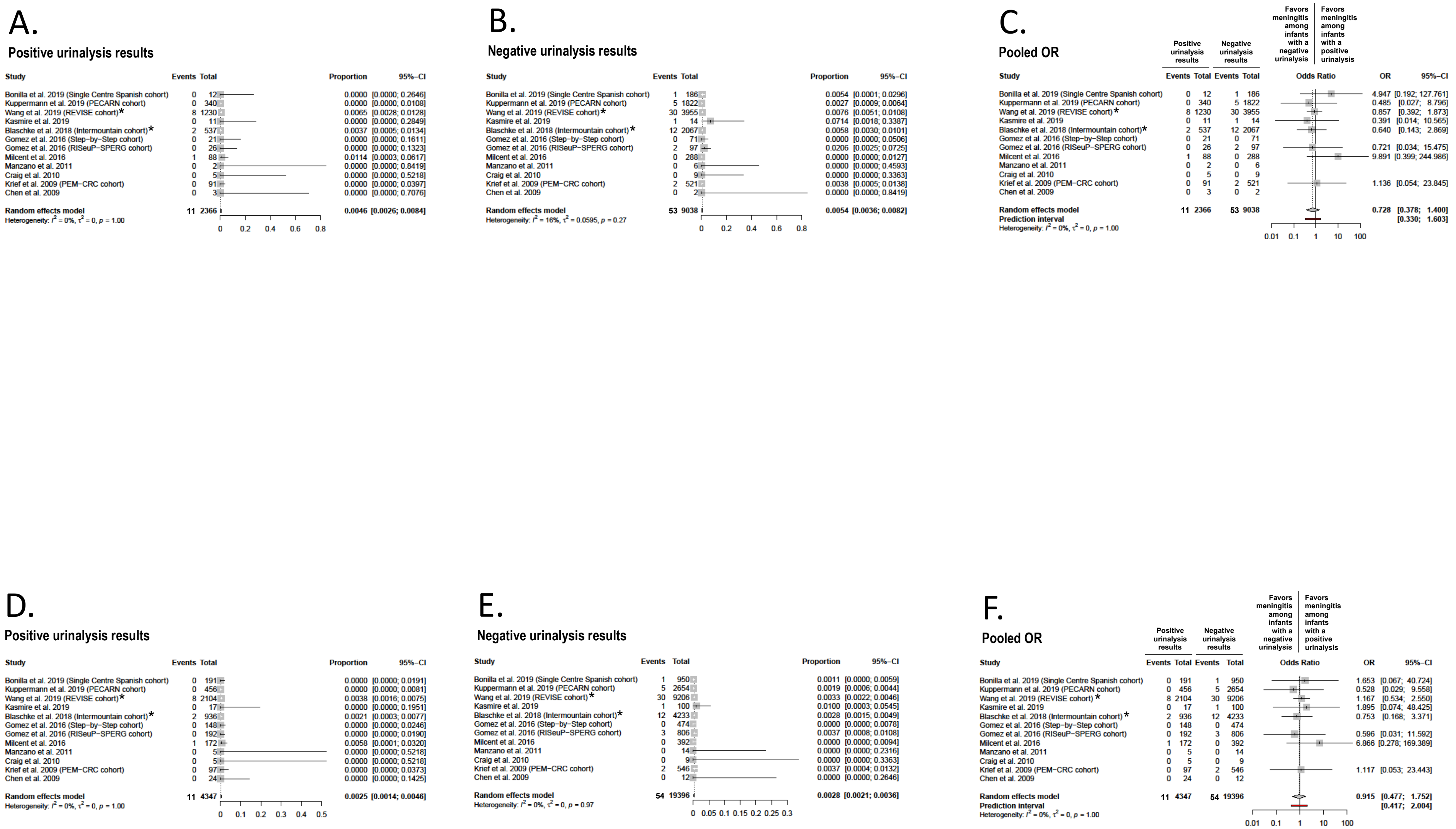

eFigure 6. Forest plots of sensitivity analysis; pooled prevalence of bacterial meningitis among urinalysis-positive infants, urinalysis-negative infants and pooled odds ratio for primary (A-C) and secondary (D-F) outcome measures, including studies with ≥7 days clinical follow-up.

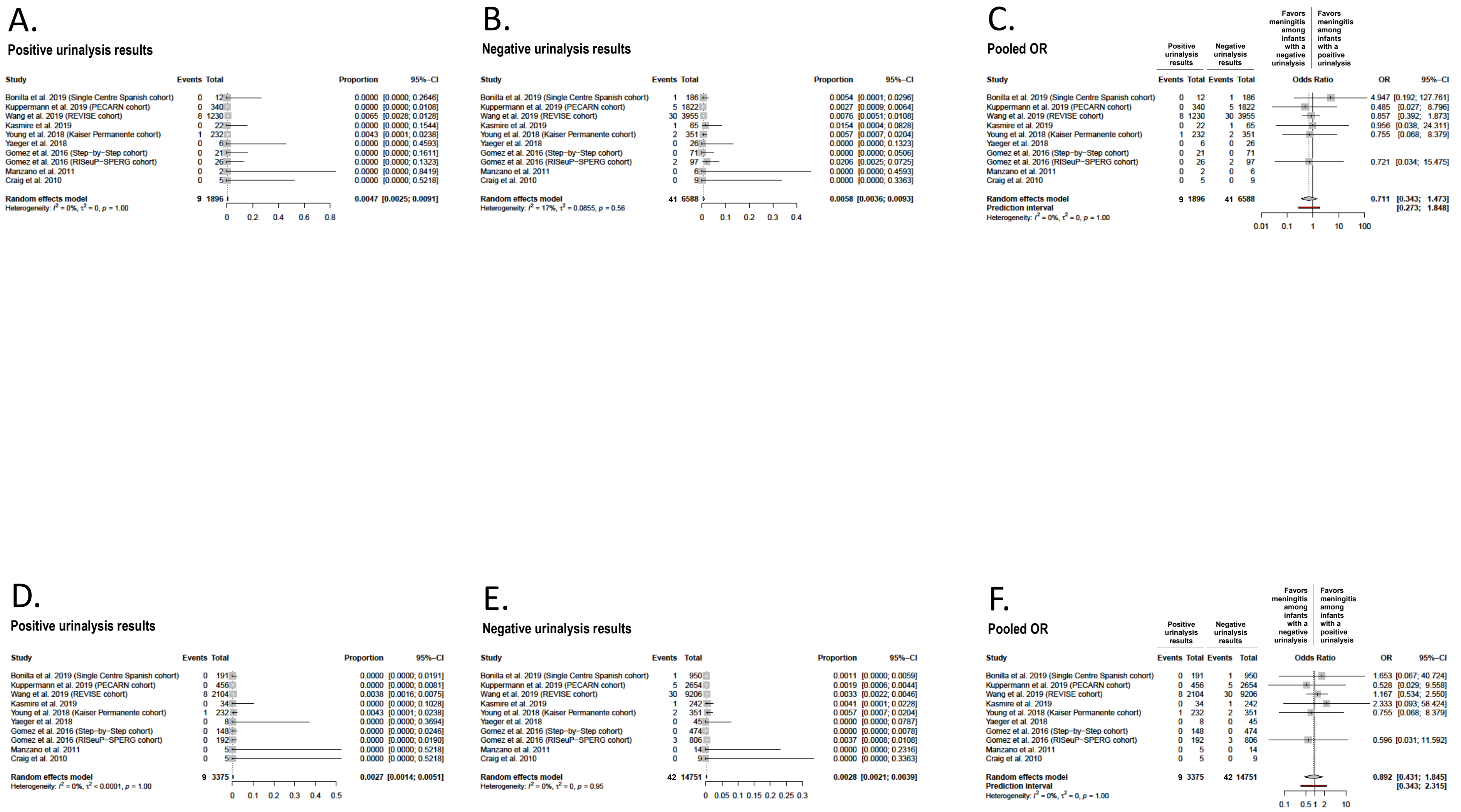

eFigure 7. Forest plots of sensitivity analysis; pooled prevalence of bacterial meningitis among urinalysis-positive infants, urinalysis-negative infants and pooled odds ratio for primary (A-C) and secondary (D-F) outcome measures, including studies with ≥30 days clinical follow-up.

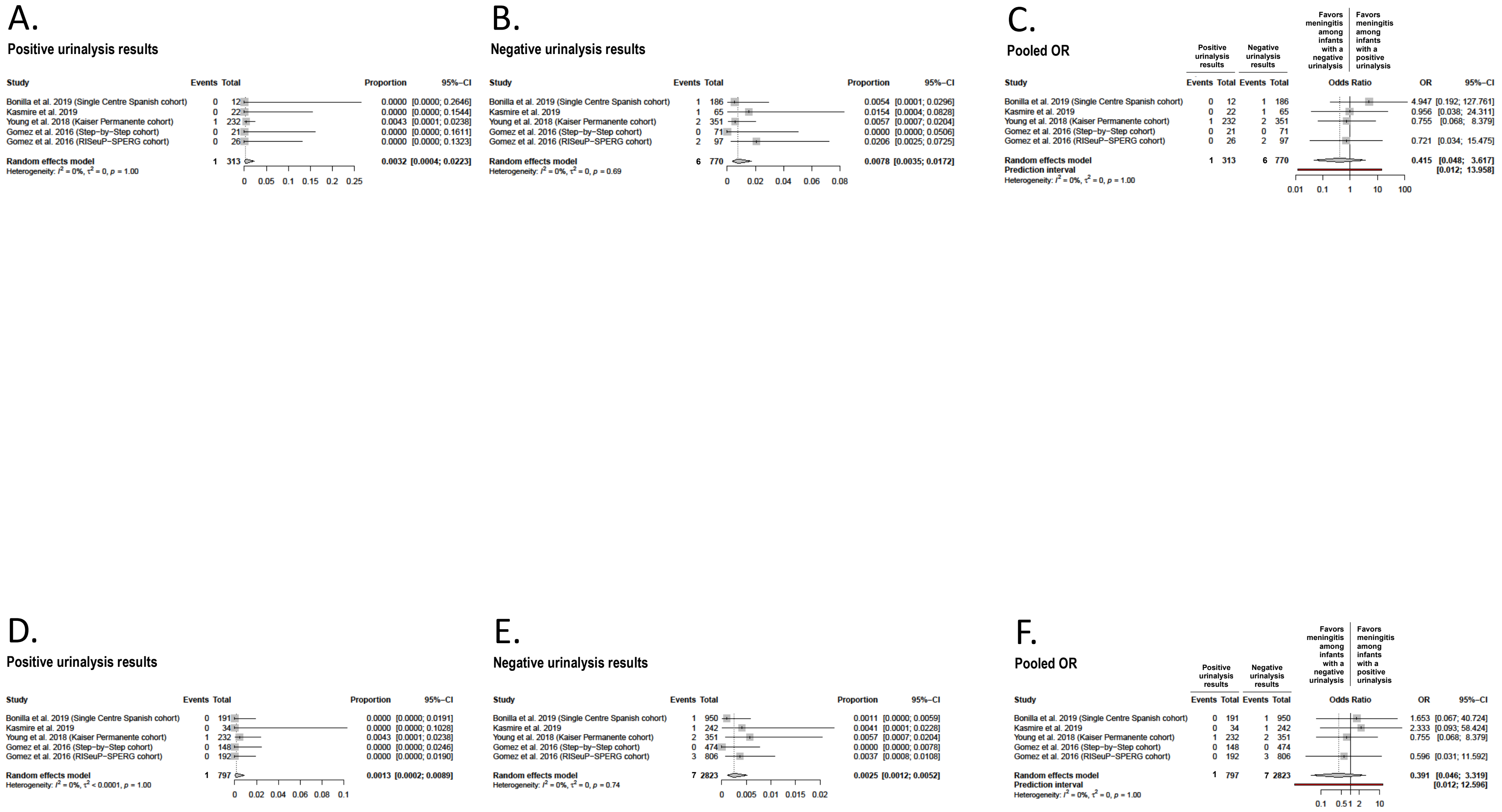

© 2021 Burstein B et al. *JAMA Network Open.*

eTable 1. Description of studies excluded after primary author contact

Reason for Exclusion	Individual Studies	Publication Year	Study Design	Enrollment Years	Country	Study Setting	Age of all Patients Enrolled	Total Number of Infants Included in Study	Follow-up Duration	Notes
No response from author	Belleau et al.	2019	Retrospective	2011-2016	France	1 Hospital	1 to 3 months	530	1 month	421 patients did not have urine dipstick or urinalysis performed
	Rabiner et al.	2019	Retrospective	2012-2014	US	2 Pediatric EDs, 1 ED	0 – 56 days	338	3 days	195 patients between 29-56 days
	Lee et al.	2018	Retrospective	2015-2017	Korea	1 ED or outpatient unit	1 to 3 months	336	Not specified	
	Carmon et al.	2017	Retrospective	2013-2014	Israel	1 Hospital	2 – 60 days	623	Not specified	386 patients between 30-60 days
	Dotan et al.	2016	Prospective	2005-2010	Israel	1 ED, 2 Hospitals	0 – 60 days	1896	Not specified	
	Diaz et al.	2016	Retrospective	2008-2012	Spain	1 Pediatric ED	0-90 days	318	Not specified	174 patients did not have LP
	Lavasseur et al.	2014	Retrospective	2003-2009	US	1 Pediatric ED	0-90 days	350	7 days	143 patients between 29-60 days. Data collected prospectively
	Nosrati et al.	2014	Retrospective	2006-2008	Israel	1 Hospital	0-90 days	401	Not specified	279 patients between 30-60 days
	Elhassanien et al.	2013	Prospective	2011	Kuwait	1 Pediatric ED	0-36 months	385	Until fever subsided	113 patients between 30-90 days
	Luaces-Cubells et al.	2012	Prospective	2008-2009	Spain	7 Pediatric EDs	1-36 months	868	28 days	325 patients under 3 months
	Martinez Planas et al.	2012	Prospective	2006-2008	Spain	1 Hospital	0-90 days	381	Not specified	

	Fouzas et al.	2010	Retrospective	2005-2008	Greece	1 Hospital	29-89 days	408	Not specified	
	Ferguson et al.	2010	Retrospective	2004-2006	US	1 Pediatric ED	28-90 days	167	2 days	79 patients between 28-59 days
	Shin et al.	2009	Prospective	2003-2006	Korea	1 Hospital	Under 3 months	221	Not specified	86 patients between 31-60 days
	Rudinsky et al.	2009	Retrospective	2002-2003	US	1 ED	0-24 months	985	Not specified	59 patients between 1-3 months. Data collected prospectively
	Pratt et al.	2007	Prospective	2002-2003	US	1 Pediatric ED	1-36 months	119	Not specified	
	Bleeker et al.	2007	Prospective	1996-2001	Netherlands	2 Pediatric EDs	1-36 months	381	2 weeks	Combined datasets from derivation and validation cohorts
	Massin et al.	2006	Prospective	2003	Belgium	1 Pediatric ED	1-36 months	376	1 year	31 patients under 3 months
	Kawashima et al.	2006	Prospective	1998-2001	Japan	1 Hospital	0-3 months	263	Not specified	67 patients between 30-60 days
No working email	Hubert-Dibon et al.	2018	Prospective	2016	France	1 Pediatric ED	7 days-5 years	1060	15 days	188 patients between 1-3 months
	Garra et al	2005	Prospective	1998-2004	US	1 Pediatric ED	0-56 days	274	Not specified	181 patients between 29-56 days
	Smitherman et al.	2005	Retrospective	1997-2001	US	1 Pediatric ED	0-36 months	705	Not specified	230 patients between 29-90 days
	Pulliam et al.	2001	Prospective	2000	US	1 Pediatric ED	1-36 months	77	Not specified	

	Herr et al.	2001	Retrospective	1999-2000	US	1 Pediatric ED	0-60 days	404	24 hours	251 patients between 29-60 days
No longer has, is unable to, or cannot provide data	Ramgopal et al.	2019	Retrospective	2006-2017	US	1 Pediatric ED	0-60 days	2930	Not specified	2058 patients between 29-60 days
	Yarden-Bilavsky et al.	2011	Prospective	2007-2009	Israel	2 Hospitals	0-90 days	1125	Not specified	581 patients between 29-60 days
	Ashkenazi-Hoffnung et al.	2011	Prospective	2005-2009	Israel	2 Hospitals	0-90 days	1584	Not specified	
	Watt et al.	2010	Retrospective	1997-2006	US	1 Pediatric ED	0-90 days	668	Not specified	285 patients between 31-60 days
	Wolff et al.	2009	Retrospective	2000-2007	US	1 Pediatric ED	6-12 weeks	1978	1 week	
	Bilavsky et al.	2009	Prospective	2005-2008	Israel	2 Hospitals	0-90 days	892	Not specified	
	Goldman et al.	2009	Prospective	2008	Canada	6 Pediatric EDs	0-90 days	257	7-10 days	193 patients between 29-90 days
	Olaciregui et al.	2009	Retrospective	2004-2006	Spain	1 Pediatric ED	4-90 days	347	Not specified	
	Maniaci et al.	2008	Prospective	2005-2007	US	1 Pediatric ED	0-90 days	234	Not specified	186 patients between 29-90 days
	Thayyil et al.	2005	Prospective	2003	UK	2 Hospitals	1-36 months	72	Not specified	
	Gajdos et al.	2005	Retrospective	1999-2000	France	1 Pediatric ED	0-92 days	315	Not specified	233 patients between 1-3 months
	Titus et al.	2003	Retrospective	1997-2001	US	1 Pediatric ED	0-56 days	348	Not specified	
Urinalysis or LP data not collected	Aldridge et al.	2017	Retrospective	2015-2016	Australia	1 Pediatric ED	0-3 months	219	48 hours	148 patients between 29-90 days

	Vujevic et al.	2017	Retrospective	2014-2015	Croatia	1 Hospital	0-90 days	181	Not specified	
	Calvo et al.	2016	Prospective	2013-2014	Spain	2 Hospitals	0-90 days	119	Not specified	
	Moldovan et al.	2015	Prospective	2013	Romania	1 Pediatric ED	7 days-12 months	90	48 hours	
	Nijman et al.	2013	Prospective	2003-2007	Netherlands/UK	3 Pediatric EDs	1 month-15 years	3201	7 days	
	Tebruegge et al.	2011	Retrospective	2001-2010	Australia	1 Hospital	0-16 years	748	Not specified	304 patients between 1-2 months
	Nijman et al.	2011	Prospective	2008-2009	Netherlands	1 Pediatric ED	1 month-16 years	1255	7 days	361 patients between 1 month - 1 year
Enrolled only infants with proven infections or abnormal laboratory tests	Aronson et al. (Pediatr Emerg Care)	2019	Retrospective	2012-2014	US	8 Pediatric EDs	0-60 days	89	Not specified	36 patients between 29-60 days
	Aronson et al. (Pediatrics)	2019	Retrospective	2011-2016	US	11 Pediatric EDs	0-60 days	543	Not specified	
	Aronson et al.	2018	Retrospective	2011-2016	US	9 Pediatric EDs	0-60 days	384	30 days	224 patients between 29-60 days
	Woll et al.	2018	Retrospective	2011-2016	US	11 Pediatric EDs	0-60 days	442	Not specified	208 patients between 29-60 days
	Velasco et al.	2017	Retrospective	2013-2015	Spain	9 Pediatric EDs	0-89 days	391	1 month	310 patients between 22-89 days
	Velasco et al.	2015	Prospective	2011-2013	Spain	19 Pediatric EDs	0-89 days	766	1 month	621 patients between 22-89 days
	Schnadower et al.	2011	Retrospective	1995-2006	North America	20 EDs: 16 pediatric EDs + 3 general EDs in USA + 1 general ED in Canada	29-60 days	1190	Not specified	1190 patients between 29-60 days

	Schnadower et al.	2010	Retrospective	1995-2006	North America	20 EDs: 16 pediatric EDs + 3 general EDs in USA + 1 general ED in Canada	29-60 days	1895	Not specified	1190 patients between 29-60 days
	Byington et al.	2004	Retrospective	1996-2002	US	1 Pediatric ED	1-90 days	1385	Not specified	Data collected prospectively

Abbreviations: ED: Emergency Department; LP: lumbar puncture.

eTable 2. Results of critical appraisal checklist for studies included in meta-analysis

Authors	Was the sample representative of the target population? (0-1 stars)	Were study participants recruited prospectively? (0-1 stars)	Sample size 0-2 stars)	Quality of descriptive statistics reporting (0-1 stars)	Selection bias (0-2 stars)	Ascertainment of positive urinalysis (0-1 stars)	Assessment of meningitis outcome (0-1 stars)	Adequacy of follow-up (0-1 stars)	Total Score
Bonilla et al. 2019[2] (Single Centre Spanish Dataset)	1	1	0	1	0	1	1	1	6
Kuppermann et al. 2019[3] (PECARN Dataset)	1	1	1	1	1	0 (bag samples included)	1	1	7
Wang et al. 2019[4] (REVISE Dataset)	1	1 (retrospective*)	2	1	0	1 (90%; 10% missing data)	1	1	8
Kasmire et al. 2019[5] (Single Study Dataset)	1	0 (2 years retrospective, 1 year prospective)	0	1	0	1	1	1	5
Young et al. 2018[6] (Kaiser Permanente Dataset)	1	0	0	1	0	0 (bag samples included)	1	1	4
Blaschke et al. 2018[7] (Intermountain Dataset)	1	1 (retrospective*)	1	1	1	0 (don't define sample collection)	1	1	7
Yaeger et al. 2018[8] (Single Study Dataset)	1	0	0	1	1	1	1	0 (7 days specified, but no data on percent lost / missed)	5
Scarfone et al. 2017[9] (Single Study Dataset)	1	0	0	1	0	0 (does not define sample collection)	1	1	4
Gomez et al. 2016[10] (European Step-by-Step Validation Dataset)	1	1	0	1	0	1	1	1	6
Gomez et al 2016[11] (RISeuP-SPERG Dataset)	1	1	0	1	0	1	1	1	6
Milcent et al. 2016[12] (Single Study Dataset)	1	1	0	1	1	1	1	1 (2 days)	7
Mintegi et al. 2014[13] (European Group Dataset)	1	0	0	1	0	1	1	0	4
Manzano et al. 2011[14] (Single Centre Canadian Dataset)	1	1	0	0 (details not specified for appearance, focus, etc.)	0	1	1	1	5
Paquette et al. 2011[15] (Single Study Dataset)	1	0	0	1	0	0 (does not define sample collection)	1	0	3
Craig et al. 2010[16] (Single Study Dataset)	1	1	0	1	0	0 (allows for voided sample, but definition of positive urinalysis is given)	1 (define in the paper as "classification verified by a final diagnosis committee")	1	5
Krief et al. 2009[17] (PEM-CRC Dataset)	1	1	0	1	1	1	1	1	7
Chen et al. 2009[18] (Single Study Dataset)	1	1	0	0	0	0 (allow for "sterile bag")	1	1	4

*analyzed with prospective studies, as all relevant covariates collected prospectively

eReferences

1. Nugent J, Childers M, Singh-Miller N, Howard R, Allard R, Eberly M. Risk of Meningitis in Infants Aged 29 to 90 Days with Urinary Tract Infection: A Systematic Review and Meta-Analysis. *J Pediatr.* 2019;212:102-110 e105.
2. Bonilla L, Gomez B, Pintos C, Benito J, Mintegi S. Prevalence of Bacterial Infection in Febrile Infant 61-90 Days Old Compared With Younger Infants. *Pediatr Infect Dis J.* 2019;38(12):1163-1167.
3. Kuppermann N, Dayan PS, Levine DA, et al. A Clinical Prediction Rule to Identify Febrile Infants 60 Days and Younger at Low Risk for Serious Bacterial Infections. *JAMA Pediatr.* 2019;173(4):342-351.
4. Wang ME, Biondi EA, McCulloh RJ, et al. Testing for Meningitis in Febrile Well-Appearing Young Infants With a Positive Urinalysis. *Pediatrics.* 2019;144(3).
5. Kasmire KE, Hoppa EC, Patel PP, Boch KN, Sacco T, Waynik IY. Reducing Invasive Care for Low-risk Febrile Infants Through Implementation of a Clinical Pathway. *Pediatrics.* 2019;143(3):e20181610.
6. Young BR, Nguyen THP, Alabaster A, Greenhow TL. The Prevalence of Bacterial Meningitis in Febrile Infants 29-60 Days With Positive Urinalysis. *Hosp Pediatr.* 2018;8(8):450-457.
7. Blaschke AJ, Korgenski EK, Wilkes J, et al. Rhinovirus in Febrile Infants and Risk of Bacterial Infection. *Pediatrics.* 2018;141(2).
8. Yaeger JP, Moore KA, Melly SJ, Lovasi GS. Associations of Neighborhood-Level Social Determinants of Health with Bacterial Infections in Young, Febrile Infants. *J Pediatr.* 2018;203:336-344 e331.
9. Scarfone R, Murray A, Gala P, Balamuth F. Lumbar Puncture for All Febrile Infants 29-56 Days Old: A Retrospective Cohort Reassessment Study. *J Pediatr.* 2017;187:200-205 e201.
10. Gomez B, Mintegi S, Bressan S, et al. Validation of the "Step-by-Step" Approach in the Management of Young Febrile Infants. *Pediatrics.* 2016;138(2):e20154381.
11. Gomez B, Mintegi S, Benito J, Group for the Study of Febrile Infant of the RiSeu PSN. A Prospective Multicenter Study of Leukopenia in Infants Younger Than Ninety Days With Fever Without Source. *Pediatr Infect Dis J.* 2016;35(1):25-29.
12. Milcent K, Faesch S, Gras-Le Guen C, et al. Use of Procalcitonin Assays to Predict Serious Bacterial Infection in Young Febrile Infants. *JAMA Pediatr.* 2016;170(1):62-69.
13. Mintegi S, Bressan S, Gomez B, et al. Accuracy of a sequential approach to identify young febrile infants at low risk for invasive bacterial infection. *Emerg Med J.* 2014;31(e1):e19-24.
14. Manzano S, Bailey B, Gervaix A, Cousineau J, Delvin E, Girodias JB. Markers for bacterial infection in children with fever without source. *Arch Dis Child.* 2011;96(5):440-446.
15. Paquette K, Cheng MP, McGillivray D, Lam C, Quach C. Is a lumbar puncture necessary when evaluating febrile infants (30 to 90 days of age) with an abnormal urinalysis? *Pediatr Emerg Care.* 2011;27(11):1057-1061.
16. Craig JC, Williams GJ, Jones M, et al. The accuracy of clinical symptoms and signs for the diagnosis of serious bacterial infection in young febrile children: prospective cohort study of 15 781 febrile illnesses. *BMJ.* 2010;340:c1594.
17. Krief WI, Levine DA, Platt SL, et al. Influenza virus infection and the risk of serious bacterial infections in young febrile infants. *Pediatrics.* 2009;124(1):30-39.
18. Chen HL, Hung CH, Tseng HI, Yang RC. Circulating chemokine levels in febrile infants with serious bacterial infections. *Kaohsiung J Med Sci.* 2009;25(12):633-639.
